# Dr. Dwight Washington Day

**Published:** 1901-12

**Authors:** 


### OBITUARY NOTES.

Dr. Dwight Washington Day, of Eau Claire, Wis., U. of B. '61, died November 19, 1901, at the age of 60 years. He was seized with apoplexy while reading a paper before the Interstate Medical Society. Dr. Day was one of the leading physicians in his section of Wisconsin. He was a native of Eagle, Wyoming Co., N. Y., and served as assistant surgeon in the 154th N. Y. Volunteers during the civil war.

